# Developing a coordinated Canadian post-secondary surveillance system: a Delphi survey to identify measurement priorities for the Canadian Campus Wellbeing Survey (CCWS)

**DOI:** 10.1186/s12889-019-7255-6

**Published:** 2019-07-11

**Authors:** Guy Faulkner, Subha Ramanathan, Matthew Kwan, the CCWS Expert Panel Group, Gaya Arasaratnam, Joan Bottorff, Alison Burnett, Peter Cornish, Rosie Dhaliwal, Matt Dolf, Tracey Hawthorn, Kandi McElary, Rachelle McGrath, Catharine Munn, Clayton Munro, Michelle Bowers, Ben Pollard, Janine Robb, James Sanford, Andrew Szeto, David Lowe, Marium Hamid, Cheryl Washburn

**Affiliations:** 1 School of Kinesiology, University of British Columbia , Lower Mall Research Station, 2259 Lower Mall, Room 337, Vancouver, BC V6T 1Z4 Canada; 2 Centre for Hip Health and Mobility, Vancouver Coastal Health Research Centre, 2635 Laurel Street, Vancouver, V6T 1M9 BC Canada; 3 Department of Family Medicine, McMaster University, 100 Main Street, Hamilton, L8P 1H6 ON Canada

**Keywords:** Mental health, Health behavior, College, University, Students, Surveys

## Abstract

**Background:**

Interventions that promote health and wellbeing among young adults are needed. Such interventions, however, require measurement tools that support intervention planning, monitoring and evaluation. The primary purpose of this study is to describe the process in developing a framework for a Canadian post-secondary health surveillance tool known as the Canadian Campus Wellbeing Survey (CCWS).

**Methods:**

Nineteen health service providers or mental health experts from 5 Canadian provinces participated in a 3-round Delphi survey by email and an in-person roundtable meeting to identify wellbeing and health behavior measurement priorities and indicators for the CCWS.

**Results:**

The final CCWS framework consisted of 9 core sections: mental health assets, student experience, mental health deficits, health service utilization/help seeking, physical health/health behaviors, academic achievement, substance use, nutrition, and sexual health behavior. Panelists generally agreed on a set of indicators, and reached consensus for at least one indicator per core section.

**Conclusion:**

This CCWS framework is the first step in developing a common surveillance mechanism tailored to the Canadian postsecondary context. Future work will include online consultation with health service providers from a broader range of post-secondary institutions, an in-person meeting with research and measurement experts to finalize survey items, and formative testing. The CCWS will play a valuable role in developing population health initiatives targeting the increasing number of young Canadians attending postsecondary institutions.

## Background

Chronic diseases (including heart disease, stroke, cancer, diabetes and hypertension) are major causes of morbidity and mortality worldwide. There is growing evidence that suggests that the initiation of major chronic diseases such as atherosclerosis, obesity, and diabetes – related to modifiable health behaviors – are emerging as early as the second and third decades of life [1–3]. Poor mental health is also one of the largest challenges facing youth, particularly as they transition from late adolescence into emerging adulthood [4, 5]. Students enrolled in postsecondary education become situated in a position of greater independence, and the behaviors acquired or reinforced during this period can help shape their future health and wellbeing [6]. Unfortunately, existing evidence suggests that this is a period of significant increases in health-risk behaviors, including increases in smoking, binge drinking and decreases in physical activity and fruit and vegetable consumption [7–9].

Population-based prevention initiatives can be cost-effective, alleviating the burden in the health-care system through future reductions of expenditures [10]. To inform intervention at the post-secondary level, a mechanism is required to assess the prevalence and correlates of mental health and health behaviors at a local level. In turn, this information may guide intervention prioritization, selection, implementation, and ongoing evaluation and program/health service refinement. Canadian colleges and universities are becoming increasingly committed to fostering student health and wellness through programming and services. In particular, at the 2015 International Conference on Health Promoting Universities and Colleges in Kelowna, British Columbia, the Okanagan Charter was established to 1) guide and inspire health promotion action, 2) generate dialogue and research on and off campuses and 3) mobilize cross-sector action to integrate health in all policies and practices [11]. To date, 12 postsecondary campuses from Canada have adopted the Okanagan Charter and accompanying calls to action: “1. To embed health into all aspects of campus culture, across the administration, operations and academic mandates. [and] 2. To lead health promotion action and collaboration locally and globally” [11]. However, adhering to these actions require surveillance tools that support planning, monitoring and evaluation.

In the absence of a coordinated Canadian system for collecting health data, some colleges and universities have been subscribing to the U.S.-based National College Health Assessment service of the American College Health Association (NCHA-ACHA). This tool, however, has a number of notable limitations. First, the survey is overly long and cumbersome with more than 300 items; second, several measures and survey tools suffer from a number of limitations [12]; third, many questions may not be of priority to Canadian stakeholders (e.g., seatbelt use) and questions in the survey reflect American health guidelines; fourth, Canadian stakeholders have expressed the need for a salutogenic tool that reflects mental health assets; and finally, the administration of the NCHA is neither comprehensive nor coordinated with Canadian research/data, thereby limiting the opportunities for institutional comparisons and to identify best practices. The NCHA is restricted to individual-level behaviors based on the cycle that each institution subscribes to, and is not ideal for institutional-level comparisons. For example, we may want to understand what programs and policies are associated with healthier student level profiles. If institutions from across Canada adopt a common surveillance tool, it becomes possible to determine over time which institutions are successful in changing health behaviors of interest. In turn, this information might pinpoint promising policies or strategies associated with such change, which can subsequently be implemented at other institutions. This also provides the capacity for quasi-experimental and natural experiments at the local, provincial or national level [13]. Creating an agile Canadian health and wellness surveillance system will serve as a critical knowledge exchange platform.

The primary purpose of this study is to describe the process in developing a framework for a contextually relevant post-secondary health surveillance tool in Canada, known as the Canadian Campus Wellbeing Survey (CCWS). The focus of this work is in developing a student-level survey to measure individual wellbeing and health behaviors. Future work will also consider approaches to capturing information at an institutional level regarding programs, policies and resources associated with wellbeing among students. We conducted a Delphi survey and consensus panel meeting to identify mental health and health behavior measurement priorities and indicators, which will help to inform the development of a 20-min survey tool. This timeframe was chosen to minimize participant dropout, and is in line with industry recommendations for online surveys [14]. A secondary purpose is to increase awareness of this evolving project among the public health community, and present a framework for which health surveillance systems may be developed in other countries with institutions adopting the Okanagan Charter.

## Methods

### Expert panel participants

All study procedures were approved by the UBC Behavioural Research Ethics Board (H18–00238). In total, seventeen health service providers (e.g., directors of wellness/wellbeing services, campus health promoters, physicians from student wellness centers) from 12 post-secondary institutions across 5 Canadian provinces were invited by email and a follow-up telephone call to participate in a Delphi survey and roundtable meeting to develop the CCWS framework. Panelists were identified by the facilitation team (GF, MK, SR) and through snowballing techniques, with consideration given to available budget, geographic distribution and expertise in student health. A student senator versed in campus mental health initiatives was also invited to provide a student voice to the panel. One final expert was invited from a provincial organization focusing on mental health initiatives within post-secondary settings. All invited panelists accepted the invitation to participate. Some panelists consulted with colleagues when completing each survey, and so the Delphi results reflected perspectives from a total of 26 members.

### Delphi survey

A three-round Delphi survey was administered by email (March–April 2018) to identify institutional priorities for core sections and indicators for a 20-min CCWS. The Delphi survey is a structured method for building consensus by administering a series of simple questionnaires to a panel of experts [15, 16]. The Delphi technique has been widely used for identifying measurement indicators in health and healthcare because it enables synthesis of knowledge from a geographically and experientially diverse group of experts with available evidence [15, 16]. Comments and feedback can be shared anonymously, prompting unbiased consideration by panel experts [15, 16]. The Delphi approach was also chosen to encourage a sense of ownership of the developing instrument which may promote future institutional uptake. The surveys and accompanying questions are available from the authors upon request. With up to two reminder emails sent the day before and on the day that each survey was due, a response rate of 100% was achieved for the three survey rounds. The correspondence process was handled by the second author.

In this Delphi survey, a list of nine core sections and accompanying indicators were generated by the facilitation team based on section headings from population surveys, including several deployed in college settings (i.e., National College Health Assessment, Healthy Minds Study, Positive Health Surveillance Indicator Framework from the Public Health Agency of Canada, and the Canadian Health Measures Survey). This list (an Excel spreadsheet) was emailed to panelists in Round 1, and panelists were asked to prioritize core sections from most to least important, and also rate indicators within each section from most to least important. A final task was to add any new indicators they perceived were absent from the list, and provide comments that could be collated and anonymously shared with the group. Panelists were given three weeks to complete Round 1.

In Round 2, the spreadsheet list was re-ordered based on mean group ratings, with the addition of core sections and indicators suggested by panelists. Separate spreadsheets were emailed to each panelist with the group mean and standard deviations for each section and indicator group, and individual Round 1 rankings. The task for Round 2 was to compare individual rankings with group means and standard deviations, revise as desired, and respond to any comments included. During Round 1, several panelists felt that indicator groups often included overlapping indicators and expressed the need to use the same rank for these overlapping items. In response, the facilitation team decided that panelists could use the same rank for overlapping items in Round 2. Panelists were given two weeks to complete Round 2. For Round 3, the spreadsheet was re-ordered as was done in Round 2 with identical instructions sent to panelists. Panelists were given one week to complete Round 3.

### Roundtable meeting: Canadian Campus Wellbeing Survey

Following all three rounds of the Delphi survey, panelists met at an in-person meeting to review Delphi findings and discuss considerations for implementing the CCWS (e.g., dealing with privacy and data sharing; improving response rates). Representatives from all 12 post-secondary institutions were present. The roundtable meeting was held May 10–11, 2018 at the University of British Columbia, Vancouver, Canada, to discuss the priority ranking results of the Delphi survey. A key meeting objective was to refine the consensus framework into a set of core sections and indicators that could be assessed within a 20-min timeframe.

Final results of the Delphi were presented to the entire group (see Table 1 for M, SD and core section rankings), and then panelists were split into three groups for roundtable sessions focusing on specific core sections. Roundtable discussions focused on three questions: a) whether the core section priority rankings were acceptable; b) within each section, prioritize specific indicators that must be included in the CCWS; and c) discuss indicators to be excluded from the core CCWS module.

**Table 1:** Core section and indicator framework for the CCWS

M(SD)	Core sections	M(SD)	Final Delphi survey indicators	Roundtable meeting indicators
1.7(0.9)	Mental health assets			
		1.6(1.5)	Resilience	^a^Resilience (e.g., control and self-efficacy, coping)
		2.3(1.1)	Psychological wellbeing	^a^Psychological wellbeing (e.g., self-rated mental health)
		3.6(2.4)	Flourishing	^a^Flourishing (e.g., life satisfaction, happiness)
		4.4(1.8)	Coping	^a^Sense of meaning or purpose
		5.1(2.4)	Self-rated mental health	
		6.1(1.6)	Stress management techniques (e.g., stress mindset)	
		7.1(1.7)	Life satisfaction	
		7.5(2.2)	Control and self-efficacy (e.g., fixed or growth mindset)	
		7.8(1.9)	Happiness	
		8.7(2.4)	Self-esteem	
		8.9(3.4)	Self-determination	
		10.9(1.3)	Sense of meaning or purpose (e.g., spirituality)	
2.0(1.1)	Student experience			
		1.3(0.5)	Sense of belonging (and conversely, social isolation and loneliness)	^a^Perceptions of campus climate (e.g., supportive learning environments, mental health support, equity and inclusion, safety, institution cares for student wellbeing)
		2.5(1.3)	Perceptions of campus climate (e.g., supportive learning environments)	^a^Overall social experience and social connectedness (e.g., meaningful connections, healthy relationships, social support)
		3.6(1.6)	Social support on campus (e.g., sense of community)	^b^Sense of belonging to any campus context (e.g., clubs, residences, sport teams); conversely, social isolation and loneliness
		3.6(1.9)	Overall social experience and social connectedness (e.g., meaningful connections, healthy relationships, social support)	^b^Negative experiences (e.g., sexism, racism, violence, discrimination)
		4.1(1.4)	Experiences of equity and inclusion (e.g., diversity); and conversely, negative experiences: sexism, racism, violence, discrimination)	^b^Financial wellbeing (e.g., access to safe and affordable housing, living arrangements)
		5.7(2.1)	Financial wellbeing (access to safe and affordable housing, living arrangements)	
		6.8(1.7)	Feelings of safety (e.g., physical and psychological)	
		7.3(2.3)	Built and natural environments (e.g., access/exposure to nature, beautiful/calm environments, learning spaces for diverse needs, time spent outdoors)	
		7.6(1.4)	Engagement in extra-curricular activities	
		8.9(1.3)	Volunteerism	
3.3(1.5)	Mental health deficits			
		1.6(1.0)	Anxiety	^a^Sources of perceived dis-stress coupled with extent of impact
		2.4(1.2)	Depression	^b^Anxiety
		3.3(1.6)	Sources of stress coupled with extent of impact	^b^Depression
		3.6(1.5)	Psychological stress	^b^Suicidal tendencies (i.e., planning, not ideation)
		4.5(1.1)	Emotional dysregulation (e.g., inability to manage emotions)	
		5.5(1.5)	Suicidal tendencies	
		6.6(0.8)	Non-suicidal self-injury	
4.1(1.0)	Health service utilization/help-seeking			
		1.6(0.9)	Knowledge and perceptions of campus mental health services	^a^Knowledge of mental health services, perceptions of campus mental health services, general sources of support (e.g., friends, family)
		1.9(0.8)	Help-seeking intentions (e.g., intentions to access other support services (e.g., career counseling, accessibility services, academic success center))	^a^Access to mental health services (e.g., stigma, timely access to counseling services/health professionals, gaps in health services on campus)
		3.8(1.3)	Use of health service facilities on campus	^b^Help-seeking intentions (e.g., intentions to access other support services (e.g., career counseling, accessibility services, academic success centre))
		4.1(1.6)	Access to mental health services (e.g., timely access to health professionals/services; gaps in health services on campus)	^b^Use of health professional services (e.g., counseling/therapy, medical professionals, emergency room)
		4.4(1.5)	Use of health professional services (e.g., counseling/therapy)	
		5.1(1.8)	Perceived stigma (e.g., self-stigma, stigma from others)	
		6.1(1.9)	Sources of health information (e.g., use of online or e-resources)	
		6.6(1.8)	Diagnosis of mental illness or condition (e.g., complex mental health diagnosis; Attention Deficit Hyperactive Disorder; Autism/Autism spectrum)	
			History of mental health services/medication	
		9.0(1.4)	Use of medication	
4.9(1.0)	Physical health/health behaviours			
		1.9(1.5)	Sleep (e.g., sleep difficulties)	^a^Sleep (e.g., sleep difficulties)
		2.6(1.3)	Physical activity	^a^Physical activity
		3.5(1.9)	Perceived health status	^a^Sedentary behavior
		4.2(1.4)	Sedentary behavior	^b^Perceived health status
		4.5(2.2)	Overall wellbeing	^b^Overall wellbeing
		4.9(1.4)	Screen time	^b^Screen time
		5.5(1.3)	Social media	^b^Social media (e.g., influence on social norms and self-perceptions)
		7.9(0.4)	Body Mass Index	
5.4(1.5)	Academic achievement			
		1.4(0.5)	Issues affecting academic performance (e.g., academic barriers)	^a^Current academic Grade Point Average (GPA) (e.g., changes in GPA, academic performance, academic comparisons with peers)
		2.1(1.2)	Overall academic experience (e.g., satisfaction with academic achievement)	^b^Issues affecting academic performance (e.g., academic barriers)
		3.2(0.7)	Experiences with faculty	^b^Overall academic experience (e.g., satisfaction with academic achievement and performance)
		4.2(0.9)	Experiences with academic support services	^b^Experiences with faculty, TA, sessional instructors
		4.8(1.6)	Current academic Grade Point Average (GPA)	^b^Experiences with academic support services
		4.9(1.0)	Academic accommodations	^b^Academic accommodations (e.g., wellbeing issues and academic concessions)
6.7(0.8)	Substance use			
		1.4(1.0)	Alcohol	^a^Alcohol
		2.5(1.1)	Marijuana/cannabis use (e.g., vaping)	^a^Marijuana/cannabis use (e.g., vaping)
		3.2(1.6)	Perception of risk regarding substance use (e.g., drinking and driving, substance use literacy, harm reduction)	^a^Drugs excluding marijuana/cannabis (e.g., opioids, study drugs (e.g., Adderall, Ritalin), use of another person’s prescription medication)
		4.6(1.5)	Drugs excluding marijuana/cannabis, use of another person’s prescription medication	^b^Perception of risk and social norms for substance use (e.g., drinking and driving, substance use literacy, harm reduction)
		4.9(2.3)	Tobacco-use (e.g., smoking, vaping)	^b^Tobacco-use (e.g., smoking, vaping)
		5.9(1.5)	Peer alcohol use	
		6.6(1.5)	Peer substance use (e.g., norms of substance use)	
		6.7(1.4)	Motivations for substance use (e.g., peer pressure)	
		8.8(2.1)	Stimulants (e.g., caffeine, medications, energy pills)	
7.6(0.6)	Nutrition			
		1.1(0.3)	Food security (e.g., access to affordable and nourishing food; alignment with eating habits and preferences)	^a^Food security (e.g., access to affordable and nourishing food, alignment with eating habits and preferences)
		2.4(1.1)	Consumption of fruits and vegetables	^b^Consumption of fruits and vegetables
		3.7(1.7)	Eating disorder symptoms (and disordered eating behaviours)	^b^Consumption of sugar-sweetened beverages
		4.1(1.7)	Weight (body) concerns/perceptions (e.g., body dissatisfaction, body image norms)	
		4.4(1.2)	Consumption of different food groups	
		5.1(1.4)	Consumption of sugar-sweetened beverages	
		6.0(1.9)	Consumption of water	
		7.1(1.1)	Availability of nutritional information	
9.0(0.3)	Sexual health behaviour			
		1.1(0.5)	Safe sex practices	^a^Safe sex practices (e.g., contraceptive use)
		2.1(0.5)	Contraceptive use	^b^Sexual satisfaction
		3.5(1.2)	Sexual activities	
		4.2(1.0)	Sexually transmitted infections	
		4.5(1.5)	Sexual orientation	
		4.8(1.0)	Sexual satisfaction	

Notes: Core sections and indicators are presented in priority sequence

Means and standard deviations refer to the final core section and indicator scores after the three Delphi rounds

^a^All three roundtable discussion groups agreed on the inclusion of this indicator in the final core survey

^b^One or two roundtable discussion groups agreed on the inclusion of this indicator in the final core survey

## Results

### Core sections and indicators

The final core section framework from the Delphi survey and roundtable meeting was nearly identical to the 9 sections generated by the facilitation team from previous surveys, with some changes to the section labels and priority sequence. Through the iterative Delphi survey, a section originally labeled “campus climate and culture” encompassed broader social determinants of health in each round, and was renamed “student experience” to capture off-campus student experiences. Another section originally labeled “eating and body image” was renamed “nutrition” to reflect that selected indicators focused on food security and eating habits most closely related to health outcomes. All other sections and labels were retained from the framework generated by the facilitation team, with minor changes with respect to priority sequence.

The final indicator framework from the Delphi survey (see Table 1, column 4) was an extensive list. This final framework was used as a prompt for discussions during the roundtable meeting. At the meeting, the primary task was to devote time to each core section and identify which indicators were critical to include, and which ones were low-priority for a 20-min core survey.

Roundtable groups used five main strategies to shorten the indicator list: 1) collapsing similar indicator concepts; 2) removing indicator concepts that are controversial and likely to be triggering for students, leading to survey dropout; 3) prioritizing indicators with known links to health outcomes; 4) prioritizing indicators that are relevant to student populations based on interests, habits or stage of development, and 5) any selected indicators must be applicable to any college or university, regardless of individual characteristics like size and location. Where there was disagreement in the smaller discussion groups regarding indicators to retain, panelists generally deferred to subject-area or measurement experts for further guidance. Overall, decisions were made based on institutional priorities, but members acknowledged that a range of considerations will be important during the next stage of CCWS question development for this surveillance tool to be applicable and useful for diverse institutions and populations (e.g., staff; colleges). Column 5 of Table 1 (‘Roundtable meeting indicators’) identifies the final indicators as a result of both the Delphi Survey and the roundtable discussions. During the roundtable, participants were invited to vote as a group as to the final inclusion of an indicator.

#### Mental health assets

All three-discussion groups agreed on the inclusion of four indicators: resilience, psychological wellbeing, flourishing and sense of meaning or purpose. Concerns were expressed that some of these terms/indicators are buzzwords that may lose currency over time. Sense of meaning or purpose, while identified as the lowest rated indicator in the Delphi survey, needed to be included within the broader context of flourishing within a post-secondary setting. Members felt that sense of meaning or purpose had fallen to the bottom of the mental health assets indicator list because it had become lumped together with spirituality (which was not perceived as critical to measure in the core survey). Consensus was reached in including sense of meaning as an indicator.

#### Student experience

The student experience section (formerly campus climate and culture) was one that grew over the course of the Delphi survey and encompassed diverse aspects of student life including social experiences, financial wellbeing and feelings of safety. For this reason, a dedicated roundtable session was set aside to discuss this section and was guided by the following questions: 1) What is meant by campus climate and culture?; 2) What specific constructs are measurable?; 3) What information would be useful for comparisons with other institutions; 4) What is already being captured in other institutional-based surveys? (i.e., National Survey of Student Engagement); and, 5) What survey questions have been useful in your own experience?

All groups reached consensus on including perceptions of campus climate, and overall social experience and social connectedness as indicators. In terms of sense of belonging, smaller group discussions revealed that this indicator should assess whether students felt connected to any campus-based groups, regardless of the specific context (e.g., are students connected to sport teams, residences, special interest groups, etc.). When it came to negative experiences, some panelists felt that this indicator should include measures of exposure to sexual harassment and abuse, as distress, mental health, physical health and substance abuse are tightly linked with experiences of sexual abuse and other forms of violence. Furthermore, many provinces and post-secondary institutions have recently developed policies and offices around these issues. Excluding this topic may lead to criticisms that the CCWS is not aligned with student and institutional concerns and priorities. Finally, although there was debate on whether it was feasible to include a complex indicator like financial wellbeing in the CCWS, questions related to safe and affordable housing was identified as an assessment priority by some institutions.

With respect to indicators that were not retained, reasons for removal from the final CCWS framework varied. For example, some members felt that assessing perceptions of the built and natural environment was important while others believed that it may be possible to capture this information from an institutional audit or other data sources. When it came to experiences of equity and inclusion, proper assessment may be beyond the scope of the CCWS. Engagement in extra-curricular activities and volunteering may not make sense for students, given their academic and work commitments. A better approach would be to look at whether students feel engaged and a sense of belonging to something beyond academics and work.

#### Mental health deficits

All groups identified sources of perceived distress coupled with extent of impact as a critical indicator. Distress, not stress, was the construct of interest, i.e., is stress impairing you? Some but not all groups retained anxiety and depression/depressive symptoms as indicators. As conversation evolved, panelists agreed that it was essential for both experiences and diagnosis of these deficits to be captured. An indicator that many believed should be included (though mindful of how it was measured and assessed) was suicidal ideation. There was, however, little consensus on this issue. For some, assessing suicidal ideation did not lead to actionable information. For others, the issue was considered to be important politically, as it receives media attention, and is something that can be taken to provincial entities or to university administration with potential funding implications.

#### Health service utilization/help-seeking

All groups prioritized two indicators: a composite of knowledge of mental health services, perceptions of campus mental health services, and general sources of support; and, access to mental health services. Access to services should, in particular, reflect any stigma related to seeking mental health support. Some, but not all groups, retained help-seeking intentions and use of health professional services.

With respect to indicators not retained in the final list, decisions were generally based on lack of applicability for a Canada-wide monitoring system, and lack of “actionable” information. For example, use of health service facilities may be best assessed locally to reflect unique aspects of individual institutions. Diagnosis of mental illness or condition, history of mental health services/medication and use of medication may medicalize the CCWS and is unlikely to provide information that campus services and programming could act upon.

#### Physical health/health behaviours

All groups prioritized indicators of sleep, physical activity and sedentary behavior. Several members felt that that perceived health status and overall wellbeing could be captured with quick single-item questions and would therefore be useful to include. There was some debate whether social media and screen time fit into the core student survey. In the end, because social media and screen time was topical for student populations, they emerged as priorities, with a view to consider questions on whether they were interfering with students’ studies and wellbeing, and how social media influenced social norms and self-perceptions. There was not much interest in body mass index, given the self-report nature of the survey and uncertainty regarding the validity of body mass index for racially diverse populations.

#### Academic achievement

All groups agreed on including an indicator of academic performance as it relates to wellbeing; but importantly, there were variations in terms of how to best capture this. Generally, there was an acknowledgement of the limitations or inherent biases associated with self-reported Grade Point Average. However, there was little consensus regarding the best way to capture academic performance with some discussion of whether perceived changes “from last semester” in Grade Point Average may be appropriate. All Delphi indicators were retained from this section by at least one group.

#### Substance use

Alcohol and marijuana/cannabis use were considered essential in assessing by all groups. Members recommended adding questions about opioids and “study drugs” to be in tune with emerging substance use concerns among post-secondary students. Perception of risk for substance use was also highlighted as an important indicator to assess, because campus programming and services may address social norms and underlying issues.

#### Nutrition

While nutrition was acknowledged as an important component of wellbeing there was also consensus that measurement challenges may restrict what could be asked within a short survey. All three groups did consider food (in)security as essential to measure. Some groups advocated for including questions on sugar-sweetened beverage consumption while others recommended assessing daily consumption of fruits and vegetables.

#### Sexual health behavior

All groups agreed on the inclusion of safe sex practices as an important indicator of sexual health behavior. Panelists agreed that it was not possible to address sexual activities and sexually transmitted diseases in the 20-min survey, and there were likely other databases (e.g., those maintained by student health services) capturing this information. There were also some suggestions that focusing on sexual satisfaction may be a more positively framed way to assess sexual health.

## Discussion

This paper describes the first step within our implementation of a comprehensive and coordinated Canadian post-secondary health surveillance system that integrates public health policy, practice, evaluation, surveillance and research. The long-term goal is to develop a comprehensive surveillance system that will enable post-secondary institutional leaders and health services to a) identify population-level estimates of health behavior and wellbeing; b) identify intervention priorities at their institutions; and c) evaluate intervention implementation. In conducting this preliminary work, we were encouraged by the level of interest and commitment of our participating panel members in moving forward in developing, and eventually collectively administering, the Canadian Campus Wellbeing Survey (CCWS). Overall, there was consensus that a common surveillance mechanism that was tailored to the Canadian postsecondary context was very much needed.

The starting point for the surveillance mechanism is creating the content of the student-level survey of health behavior and wellbeing. Results showed consensus in the core section priority rankings, and general agreement in the top-rated indicators from each core section to be included in the CCWS. Within each core section, consensus was reached for at least one indicator. Highest levels of agreement emerged for the top-rated mental health assets section, as all discussion groups independently agreed on four priority indicators to include in the final framework. In particular, there was demand for the CCWS to move away from a mental illness model to one that operationalizes mental health as symptoms of positive feelings and positive functioning in life [17]. This is in contrast with the most commonly used tool in Canada, the NCHA, and also the more recent World Health Organization World Mental Health Surveys Initiative International College Student Project (WMH-ICS), which is exclusively focused on mental disorders [18, 19]. The concepts of flourishing and resilience were considered central to the CCWS.

There was also consistent recognition of the bi-directional relationship of wellbeing and health behaviors, and the continued importance of monitoring the most consistent behavioral risk factors for premature chronic disease including tobacco use, physical activity and sedentary behavior, and binge drinking. Sleep emerged as a health behavior that was considered essential to assess as well. Given emerging trends in Canadian population guidelines and research looking at movement across a continuum from sleep to sedentary behavior, and light, moderate and vigorous activity, [20–23]. assessing these movement behaviors will ensure alignment with the new adult guidelines under development.

Most debate was reserved for capturing and defining the parameters for measures related to campus climate and student experiences. Clearly, measuring a sense of belonging or social connectedness was consistently important for panel members, and in line with theoretical frameworks such as Self-Determination Theory, [24]. that highlight the importance of connectedness for wellbeing. There is also growing evidence that a feeling of connectedness itself protects against engagement in health-risk behaviors [25]. There was polarity when thinking about measuring experiences of equity and inclusion (e.g., diversity); and conversely, negative experiences including sexual assault, racism, or discrimination. Discussion groups considered how much overlap there should be with other existing institutional surveys that could be deployed to more adequately examine these sensitive issues.

In developing the CCWS, a decision was made to develop a 20-min core survey that is relevant to all Canadian postsecondary settings and potentially applicable for faculty, staff and campus neighborhood residents. This will necessitate some difficult decisions in focusing on the top-ranked indicator(s) in each core section. This is made easier by harmonizing the CCWS with other complementary surveys conducted in Canada. For example, there was less interest in a detailed exploration of alcohol and drug use within the CCWS given the recent Health Canada initiative to develop a postsecondary drug and alcohol survey in collaboration with the Postsecondary Education Partnership- Alcohol Harms (PEP-AH) [26]. More detailed exploration of issues regarding sexual violence and campus climate in general could be assessed through the EAB Climate Survey [27]. As the CCWS grows, there is the potential to develop additional modules (e.g., sexual health) that can be included in concert with the core module depending on institutional interest. In other words, the CCWS will be flexible and able to accommodate additional questions for individual institutions. This flexibility will be important to address emerging concerns such as opioid use on particular campuses or the rapidly changing nature of social media. While not in the current scope of our work, the CCWS is also designed so it could be implemented for assessing the wellbeing of staff, faculty or campus neighborhood residents.

The next stage of developing the CCWS will involve online consultation with health service providers from a broader range of post-secondary institutions through the Canadian Association of College & University Student Services (CACUSS) (e.g., colleges, polytechnic institutes, rural and northern locations), to solicit additional feedback on the proposed CCWS framework. This will be followed by an in-person meeting with research and measurement experts to identify and finalize CCWS items. The CCWS will use measures with demonstrated reliability and validity that are consistent with national surveillance tools. Formative testing at pilot university and college sites will take place early 2019 to ensure that the survey is relevant to, understood by, and convenient for post-secondary students to respond to, and can be completed within a 20-min timeframe. The aim is to deploy the CCWS at select post-secondary sites in the 2019–2020 academic year.

The methodological approach we adopted was successful as a first step in developing a common surveillance mechanism tailored to the Canadian postsecondary context. It also created a space for collegial discussion of the importance of creating a national platform for knowledge exchange and evolving a stronger community of practice. There were some limitations to the approach. First, given budgetary and time constraints, participants were selected through personal contacts of the research team and were individuals who had expressed interest in developing a new student level survey for use in Canada. It is not known how different perspectives may be from other institutions not represented. Further consultation regarding the framework is planned throughout 2019. Second, consensus was not always met on some topics for inclusion. An example was perceptions of the built environment. In such cases where there was no clear consensus, the research team erred on the side of caution in excluding the indicator with a view that future modules could be created to assess topics of interest to institutions. The goal of creating a twenty-minute survey was central to such decisions. Finally, the Delphi Survey was not anonymous as the second author was aware of individual responses throughout the survey process. At the same time, indicator rankings and comments were collated and shared anonymously with the group.

In parallel with the work on the CCWS is the planned creation of a national standard for post-secondary student mental health to support student success on campuses across Canada. The Mental Health Commission of Canada (MHCC) will be leading the project to establish the standard in collaboration with other Canadian organizations [28]. Most critically, there are plans to develop a formalized audit tool for institutions to assess progress toward meeting those standards, which would be complementary to the individual-level CCWS. For example, this institutional-level tool can be linked to the CCWS to explore how institutional variability in policies and programs may explain variability in student-level wellbeing outcomes. Importantly, these tools will be essential to the evaluations of campus-based programs, policies, and initiatives implemented based on the Okanagan Charter and its Call for Action. Bringing together student and institutional-level data has the potential to create a powerful knowledge exchange platform for Canadian institutions to identify what works best, for whom, and under what circumstances – and such evaluative mechanisms should be a priority for any institution adopting the Okanagan Charter.

## Conclusion

Much work remains in finalizing the CCWS, piloting its administration, and addressing the pragmatic institutional concerns related to sensitive issues such as data sharing, reporting, and management. Developing a common Canadian surveillance and knowledge exchange system at the postsecondary level is an ambitious vision, but we believe integral to population health initiatives targeting the increasing number of young Canadians attending postsecondary institutions.

## Data Availability

The datasets used and/or analyzed during the current study are available from the corresponding author on reasonable request.

## Abbreviations

CCWS: Canadian Campus Wellbeing Survey
NCHA: National College Health Assessment
